# Indirect Monitoring of Frequencies of a Multiple Span Bridge Using Data Collected from an Instrumented Train: A Field Case Study

**DOI:** 10.3390/s22197468

**Published:** 2022-10-01

**Authors:** Abdollah Malekjafarian, Muhammad Arslan Khan, Eugene J. OBrien, E. Alexandra Micu, Cathal Bowe, Ramin Ghiasi

**Affiliations:** 1 Structural Dynamics and Assessment Laboratory, School of Civil Engineering, University College Dublin, D04 V1W8 Dublin, Ireland; 2 School of Civil Engineering, University College Dublin, D04 V1W8 Dublin, Ireland; 3 Department of Civil Engineering, Trinity College Dublin, D02 PN40 Dublin, Ireland; 4 Iarnród Éireann Irish Rail, Technical Department, Engineering & New Works, Inchicore, D01 V6V6 Dublin, Ireland

**Keywords:** multi span bridge, drive-by, natural frequency, indirect, bridge monitoring

## Abstract

In this paper, a field study is carried out to monitor the natural frequencies of Malahide viaduct bridge which is located in the north of Dublin. The bridge includes a series of simply supported spans, two of which collapsed in 2009 and were replaced. The replaced spans are stiffer than most of the others and these differences resulted in higher natural frequencies. An indirect bridge monitoring approach is employed in which acceleration responses from an instrumented train are used to estimate the natural frequencies of each span of the viaduct showing the locations of the two replaced spans with higher stiffness. For the indirect approach, an Ensemble Empirical Mode Decomposition (EEMD)-based Hilbert Huang Transform (HHT) technique is employed to identify the natural frequency of each span. This is carried out by analysing the Instantaneous Frequencies (IFs) from the calculated intrinsic mode functions. The average of the IFs calculated using 41 runs of the instrumented train (with varying carriage mass and speed for each run) are used to estimate the natural frequencies. To assess the feasibility of the indirect approach, a bespoke set of direct measurements was taken using accelerometers attached successively on each span of the viaduct. The free and forced vibrations from each span are used to estimate the first natural frequencies. The frequencies obtained from drive-by measurements are compared to those from direct measurements which confirms the effectiveness of indirect approaches. In addition, the instantaneous amplitudes of the drive-by signals are used to indicate the location of the stiffer spans. Finally, the accuracy and robustness of the indirect approaches for monitoring of multi span bridges are discussed.

## 1. Introduction

Bridges are arguably the most critical structures in transportation infrastructure. They require monitoring to detect local and global damage caused by several reasons such as bridge strike, fatigue, loading and scour [1,2,3,4,5]. Historically, bridge collapses result in traffic disruptions and hindrance to the public services that, e.g., resulted in a case of the Caprigliola bridge collapse (2020) [6], and the Malahide estuary viaduct failure (2009) [7]. Given their central function in transport infrastructure, researchers have been seeking efficient and reliable ways to identify bridge damage before it becomes catastrophic [1,2,8]. Traditionally, a bridge is monitored directly by installing sensors to obtain responses to load. This approach uses direct on-bridge responses to infer mechanical and dynamic properties of the structure and compare them with healthy baseline values to identify any damage present [9,10]. While this type of monitoring has been seen to be effective, it requires a distribution of sensors along the length of the bridge, that consumes a lot of cost and labour. For longer multiple span bridges in particular, traditional methods of monitoring can become difficult and costly, since they require more sensors [4,11,12].

Bridge dynamic responses to ambient, forced, or free vibrations, are often used for bridge damage detection [3,5,13,14]. Accelerations contain damage-sensitive features, such as natural frequencies, which make them a potential parameter for developing damage detection methods using signal processing techniques. Many studies have estimated natural frequencies from acceleration responses obtained using numerical models [15] as well as bridge field measurements in a form of free vibration [16], forced vibration [14,17], or ambient vibration [18]. The natural frequencies are considered as some of the key damage detection features for these studies and have provided reasonable results for the detection of both local [19,20] and global damage [11,21,22,23]. Although, natural frequencies are not considered to be ideal, because of their low-sensitivity to damage [3,24], changes in the natural frequencies and modal properties have been used to detect scour damage in multiple span structures [5,11,25,26] and longer span bridges [27]. The significant impact of a scour-hole at the foundation has been shown to affect the entire bridge response [11,28]. In numerical [11], and laboratory setups [26] for multiple span bridges, the impact of damage on each span’s natural frequency is found to be reasonable, that can be understood using advanced frequency-domain-based [29] or mode-shape-based methods [4,5]. Although these studies provide a good understanding of damages and structures, validation of these techniques in the field requires distributed sensors in a large quantity to cover the entire structure. For that reason, the field measurement of multiple span bridges poses a challenge for researchers to develop practical approaches for inspection.

Indirect or drive-by techniques have recently gained interest among Structure Health Monitoring (SHM) researchers because of the ease with which dynamic responses can be measured [2,30,31,32]. This technique extracts features of the structure from the dynamic responses measured in a moving body interacting with it, such as a truck or train. Many existing drive-by approaches focus on extracting bridge dynamic features such as natural frequencies and modal parameters and study the changes in them due to, e.g., crack damage on the deck [33], scour damage [26] or bearing seizures [14]. A key challenge in indirect monitoring techniques is the separation of bridge-related frequencies from vehicle frequencies in the measured response. This becomes difficult if the vehicle and the bridge frequencies are in a close proximity [34] when mode mixing can occur [35]. Vehicle frequencies tend to dominate the measurement, making it more difficult to extract bridge frequencies from the response. To address this problem, researchers use signal optimization and advanced processing techniques [20,24,36], that improve the resolution of bridge-related frequencies in the measured vehicle response. Gul et al. (2021) use an inverse filtration approach on smartphone accelerations, measured inside a vehicle, to address the mixing of vehicle and bridge frequencies. They test the approach in a laboratory setup [37], and using two real life bridges [38]. Dhakal and Malla [39] employ indirect measurements to estimate railway bridge natural frequencies in an experimental setup and compare their results with a finite element (FE) model of the bridge. Studies on extracting bridge frequencies from drive-by measurements have used many numerical techniques: Hilbert Transform (HT) [40], moving average filtration [23,33], wavelet analysis [11], signal decomposition techniques [41], and frequency-independent underdamped pinning stochastic resonance [36]. Although these techniques have shown effective results in the numerical studies, and some single span real bridges, their implementation on multiple span bridges still requires more testing and research.

For signals in the time or frequency domain, several signal processing techniques have been proposed for the extraction of features from the acceleration responses. These include short-term Fourier transform [42], wavelet transform [11], singular value decomposition [43], kernel time-frequency representation [44] and more. Each one has its own merits and demerits. Signal decomposition is one of the commonly used approaches for separating vehicle or bridge frequencies from a drive-by signal. One of the methods of signal decomposition is the Hilbert Huang Transformation (HHT) [45] that analyses non-linear and non-stationary signals by applying the HT. The original signal is decomposed into Intrinsic Mode Functions (IMFs) that are extracted/separated using a sifting process called Empirical Mode Decomposition (EMD) [46]. The HHT of an acceleration response identifies a number of Instantaneous Frequencies (IFs) that can be presented as IMFs in time and frequency domains [45,47]. For the case where vehicle and bridge frequencies are close together, the phenomenon of mode mixing occurs that makes it difficult to separate these frequencies into individual IMFs using EMD [48]. For that problem, Wu and Huang (2009) propose a noise-assisted analysis approach called Ensemble Empirical Mode Decomposition (EEMD) [35] that can address the mode mixing problem of the EMD [35,49]. Zhu and Malekjafarian [50] have recently proposed an EEMD based drive-by approach for estimating bridge natural frequencies from vehicle responses. They suggest that EEMD is less sensitive to measurement noise and provides higher accuracy of bridge first natural frequency from the vehicle responses. Although the method has shown promising results in numerical and laboratory setups, a field verification would help to confirm its efficacy.

In this paper, an improved HHT (using EEMD) is applied to the acceleration measurements from an instrumented train to estimate the natural frequencies of the Malahide Railway Viaduct in Ireland, which consists of 12 simply supported spans. To the best knowledge of the authors, this is the first time that a drive-by technique is employed to estimate the fundamental frequency of each span of a multi span viaduct bridge in field. To validate the drive-by technique, a direct test is also carried out to find the true natural frequency of each span. This bridge experienced a collapse of two spans and one pier foundation in 2009 due to scour. These spans were replaced with stiffer elements [7,51] which has resulted in a change of their natural frequencies. In this paper, an instrumented railway carriage of an in-service passenger train collected accelerations as it traversed repeatedly over the viaduct on the Dublin–Belfast railway track. The train drive-by data is decomposed using EEMD to measure the IMFs, and HHT is applied to measure the IFs from each measured IMF. The IFs corresponding to the bridge natural frequencies are estimated and compared with the natural frequencies measured directly from the free and forced vibrations of each span. The drive-by measurements of the instantaneous frequencies are compared with the direct measurements taken on the bridge. 

## 2. Theoretical Background 

### 2.1. Ensemble Empirical Mode Decomposition (EEMD) Method

In the EMD process, the acceleration signal is decomposed into multiple IMFs, which are oscillatory functions with varying energy amplitude and frequency [46]. The EEMD approach is introduced by Huang and Wu [52] that addresses the issues relating to mode mixing and high signal noise. The EEMD is an iterative process, where an adaptive white noise *n*(*t*) signal is introduced in the original acceleration signal *x*(*t*). Each iteration (*i*) is given by Equation (1). The added noise provides a uniformly distributed reference frequency and IMFs associated with a series of random and uncorrelated noise signals.

(1) $$x_{i}\left(t\right)=x\left(t\right)+n_{i}\left(t\right)$$

Using the noisy version of the signal, *x_i_*(*t*), decomposition is carried out repeatedly and the ensemble means of the IMFs are the final results. The level of added noise and the number of iterations can affect the final IMFs from EEMD. Hence, finding their optimum values for each case is important for the accuracy of the results [35,52]. With low added noise in the EEMD process, the decomposition will not be effective because of insufficient changes in the extremes. On the other hand, excessively high added noise results in the calculation of a higher number of redundant IMFs. For example, Aied et al. [49] use the EEMD process for the measurement of bridge stiffness using accelerations and test the results with different levels of added noise to the process. They state that in order to separate mixed modes in a signal where low frequencies dominate, a higher amplitude of noise should be used and vice versa. 

### 2.2. Hilbert Huang Transformation (HHT)

The HHT is a two-step process consisting of: (1) decomposition of a signal into multiple IMFs (*C_r_*(*t*)) with varying frequency and amplitude using EMD or EEMD, and (2) application of the HT (given in Equation (2)) to the measured IMFs to calculate the instantaneous frequencies (IFs) [47]. *H*(*C_r_*(*t*)) represents the HT of the *r*th IMF and can be defined as:

(2) $$H\left(C_{r}\left(t\right)\right)=\frac{1}{\pi }P\int_{-\infty }^{\infty }\frac{C_{r}\left(\tau \right)}{t-\tau }d\tau$$

where *t* represents time and *P* represents the Cauchy principal value of the singular integral. The HT results can be grouped for each IMF to form an analytic signal *a*(*t*) as a complex function:

(3) $$a_{r}\left(t\right)=C_{r}\left(t\right)+jH\left(C_{r}\left(t\right)\right)=b_{r}\left(t\right)e^{j\theta_{r}\left(t\right)}$$

where *j* is imaginary unit, $b_{r}\left(t\right)$ and $\theta_{r}\left(t\right)$ represent instantaneous amplitude and phase functions of the *r*th IMF, respectively. By differentiating the phase functions with respect to time, the IFs can be calculated [47]. A Hilbert Spectrum can then be plotted using the real part of the IFs in the time and frequency domains. 

### 2.3. EEMD Based HHT for Multiple Span Bridge Frequency

In this paper, EEMD based HHT is used to estimate the natural frequencies of a multiple span bridge using train accelerations. In the drive-by signal, mode mixing creates a challenge for researchers to estimate the natural bridge frequencies [38]. For that reason, researchers use filtration or other mathematical approaches to intensify the bridge natural frequencies relative to other signal frequencies. For the case of a multiple span bridge, the drive-by signals are more complicated since the bridge frequencies change from span to span and mode mixing becomes more significant, especially when the spans are of equal length (and therefore frequency). For this scenario, an EEMD based HHT of the drive-by acceleration data seems more practical and feasible. With EEMD, the HHT of the IMFs provides instantaneous frequencies (relating to natural frequencies) in the time domain. This is significant, as any change in the bridge IFs can represent a change in the stiffness or mass at that location (span) where the instrumented carriage is currently traversing. This method is useful for detecting changes in the frequencies where the bridge may have experienced scour (caused by a flooding event) or any global change in the stiffness or mass conditions. In this paper, the IFs are estimated using the EEMD based HHT of acceleration signals from the instrumented train. Then, an average of these IFs is calculated for each span. The accuracy of the inferred bridge natural frequencies is assessed by comparing the average of the IFs with the actual bridge frequencies measured directly from free vibration data. 

## 3. Field Measurements 

### 3.1. Malahide Viaduct UBB30 Ireland

**Figure 1:**
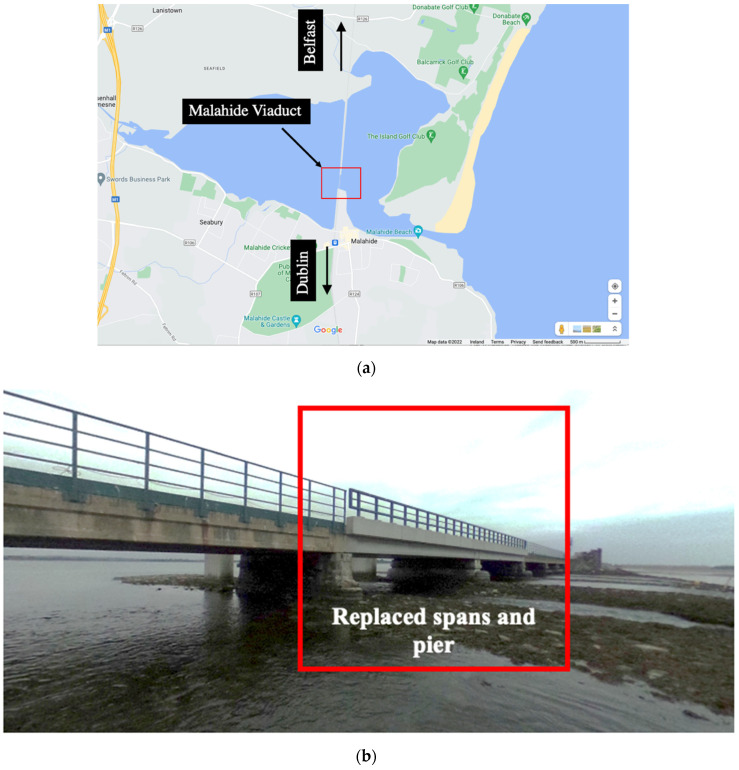
(**a**) Location of the Malahide Viaduct (12-span bridge) (taken from Google Maps), and (**b**) photograph of the viaduct with box showing the location of the replaced spans, Span 4 and Span 5, and the pier) (courtesy of Irish Rai).

**Figure 2:**
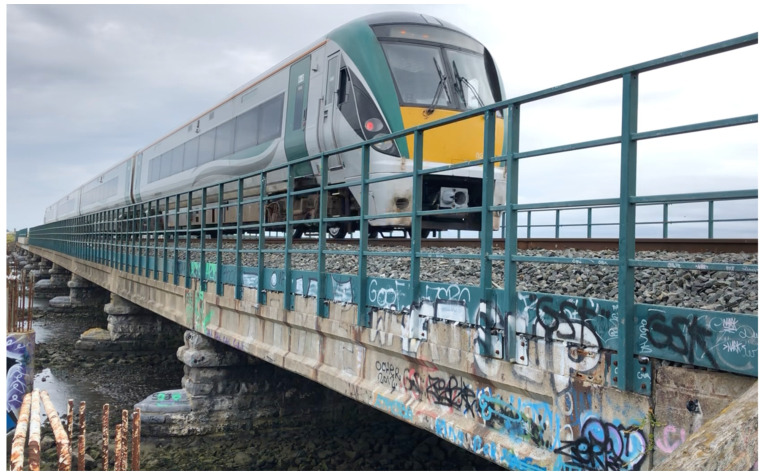
The full view of the bridge with a passing train.

The Malahide Viaduct is a 12-span bridge, constructed in 1844 over the Broadmeadow Estuary, Dublin, Ireland (Figure 1). It contains a double railway track that serves the Dublin–Belfast railway line and freight trains. In 2009, two of its spans (Spans 4 and 5, as shown in Figure 2) and Pier 4 collapsed due to scour caused by a flash flooding event [51]. After the event, the viaduct has been rehabilitated and monitored to avoid the risk of soil erosion and damages caused by increased loading conditions from the passing trains [7]. The collapsed spans and pier were replaced with stiffer spans and new pier foundations were installed with micropiles [53,54] (see Figure 1b with box showing the replaced spans and pier). The total length of the viaduct is almost 175 m, and the width is approximately 9.0 m. The first two spans at both ends (Spans 1, 2, 11 and 12) are 12.2 m long, and are shorter than the remaining, inner spans (Spans 3–10), which are 15.85 m long. The spans are made from precast concrete beams with an in situ deck, and the original piers from cut stone masonry. Pier 4 (replaced after the collapse in 2009) is made from in situ reinforced concrete with a precast bearing shelf [7]. The entire viaduct bridge when a train is passing over it, is shown in Figure 2.

### 3.2. Direct Measurements

**Figure 3:**
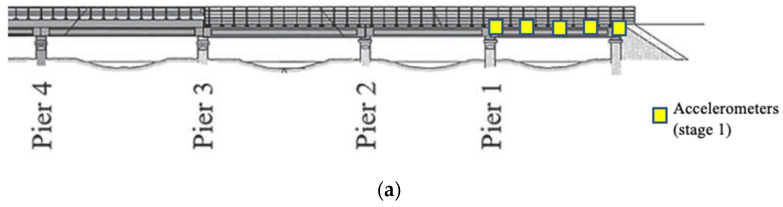

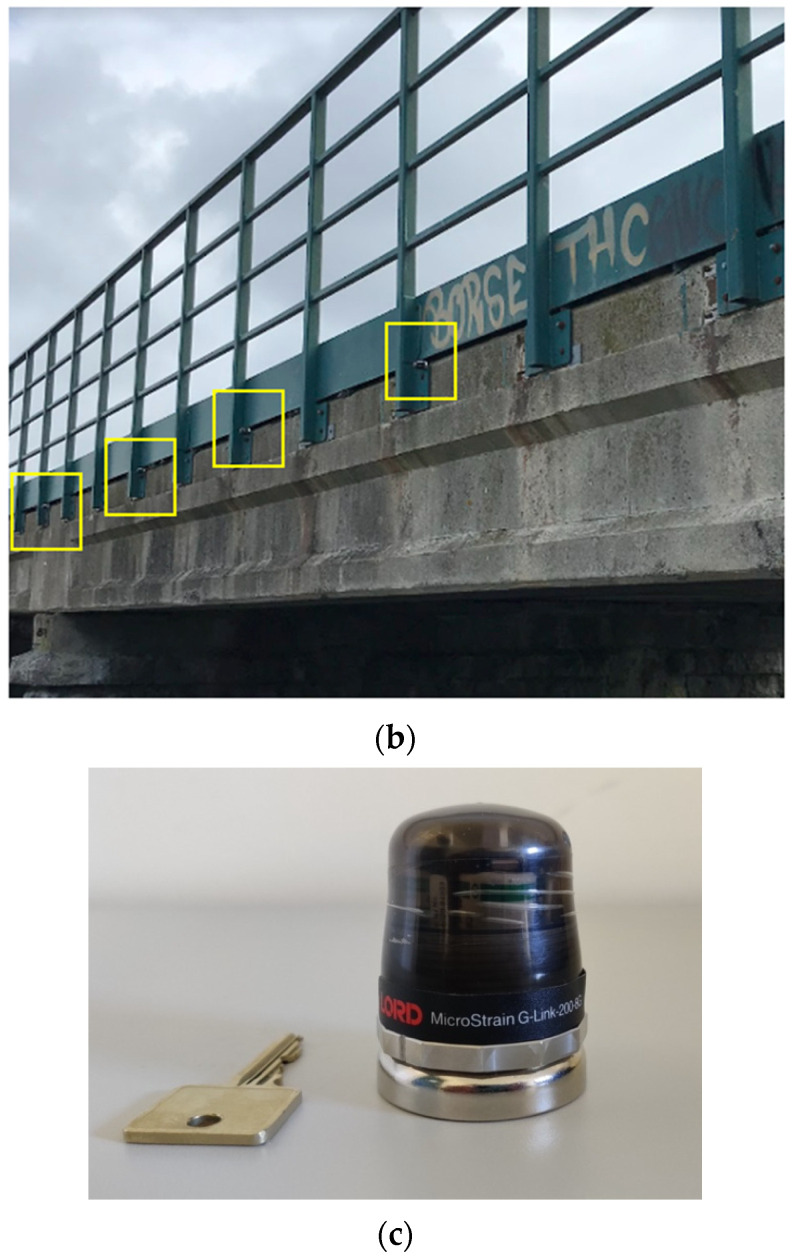
Layout of sensor installation for a typical stage: (**a**) plan of layout; (**b**) detail at West side (yellow boxes show the locations of installed sensors); (**c**) photo of the sensor used in direct measurements.

To provide a baseline to compare with the drive-by measurements, the natural frequencies of each span of the viaduct are measured directly by installing accelerometers on the bridge. Five triaxial wireless MEMS accelerometers (LORD MicroStrain G-Link 200, MicroStrain, Inc., Williston, VT, USA, see Figure 3c) were used to record the deck free vibrations of each span. The free vibrations were measured following excitation caused by the passing trains. Typically, the recordings were continued for three seconds after the passage of each train.

The direct measurements were carried out in twelve stages. In each stage, a different span was instrumented and monitored using five accelerometers placed at the points where the West side handrail uprights are connected to the concrete beam (Figure 3b). The sensors were installed near the piers at each end, midspan, the first and the third quarters (Figure 3a). After each measurement stage, the five accelerometers were moved to the next span, eventually covering all twelve spans of the viaduct. Magnetic mounting bases were used to attach the wireless accelerometers which were Lithium battery powered. Vertical accelerations were recorded at a scan rate of 256 Hz. 

**Figure 4:**
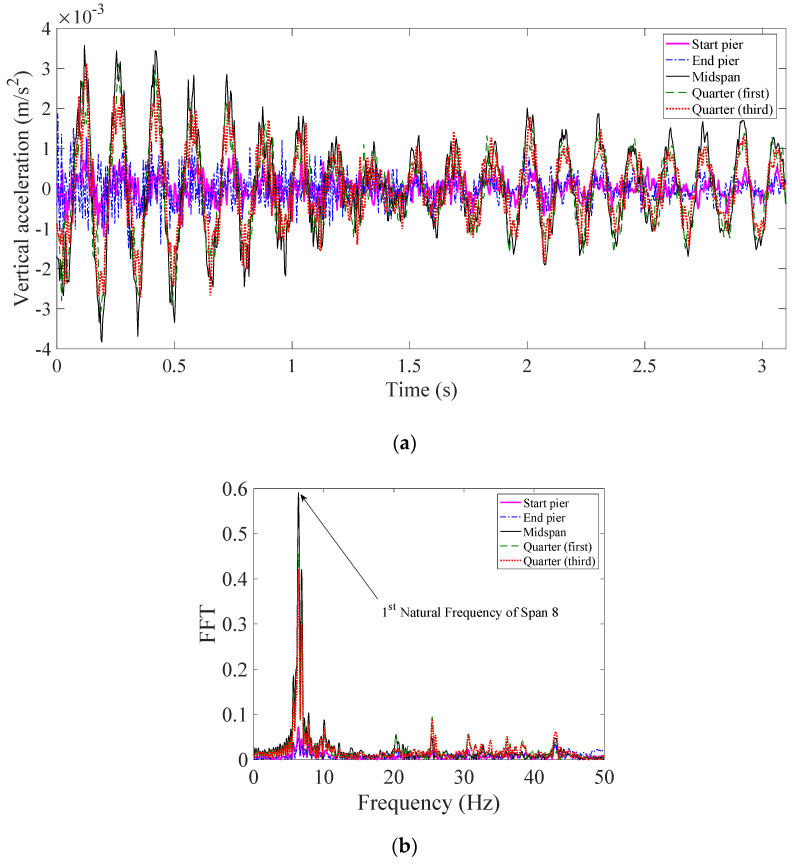
Free-vibration response of Span 8: (**a**) in time domain, and (**b**) in frequency domain.

**Figure 5:**
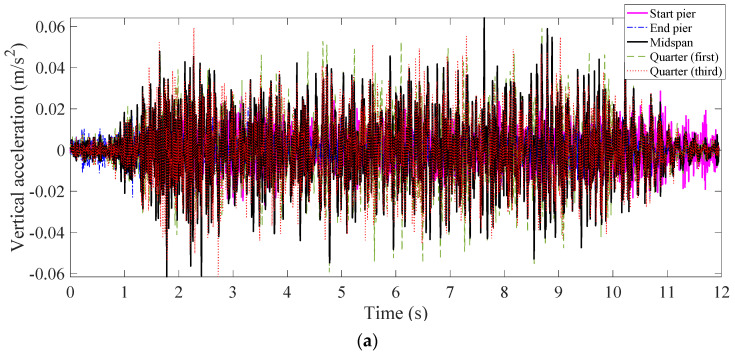

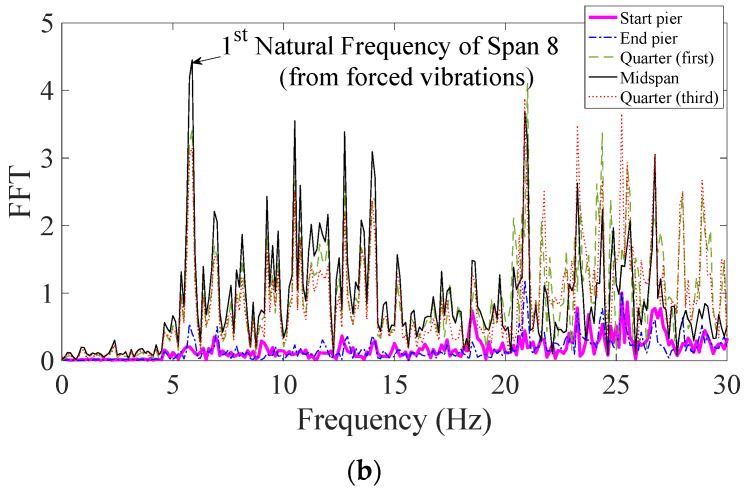
Forced-vibration response of Span 8: (**a**) in time domain, and (**b**) in frequency domain.

The first natural frequency of each span of the Viaduct was calculated using a Fast Fourier Transformation (FFT) of the free and forced vibration data. For each span, the forced and free vibrations after three passing trains were used to estimate the first natural frequency. An example of the vibration signals measured at five locations of span 8 (one of the existing/old spans with longer length) and their FFTs following a train crossing, are shown in Figure 4 and Figure 5 for free and forced vibrations, respectively. In this example, the bridge is excited by a passing freight train (consisting of 11 carriages) on a Dublin to Belfast route. From comparing the forced and free vibration signals, it can be seen that the later shows more frequencies (containing both the vehicle and the bridge frequencies) in a range higher than the bridge fundamental frequency. The first peak in the frequency domain, as shown in Figure 4b, corresponds to the first natural frequency of the bridge, which was evident in the data from all five sensors at Span 8. Similarly, the bridge frequencies are also estimated from the forced measurements. The first natural frequency of each span (averaged from free and forced vibrations caused by three passing trains) is summarized in Table 1. From this table, it can be seen that (1) the shorter spans have higher first natural frequency, as expected, (2) the first natural frequencies of the replaced spans (Span 4 and 5) are higher than the other spans of equal length (Span 3 and Spans 6–10), (3) the frequencies estimated from the forced vibrations are lower than the frequencies from the free vibrations, which may be a result of an added mass of the passing train. This confirms that the replaced spans are different than the other ones, due to their higher stiffness to mass ratio.

### 3.3. Instrumented Train and Its Properties

**Figure 6:**
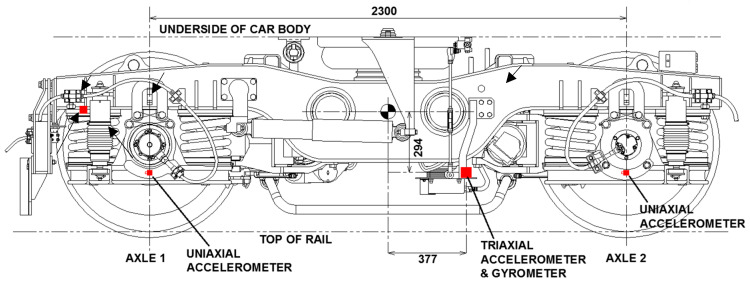
Layout of the sensors on an instrumented bogie of the train (dimensions in mm) taken from [55].

A five-carriage train is instrumented using multiple accelerometers and a Global Positioning System installed at the first bogie of the leading carriage [54]. The layout of the sensors is shown in Figure 6 [7]. Vertical bogie accelerations were recorded over a 5-week period in 2016 as the train traversed the viaduct 41 times. More details of the instrumentation and the test are presented in [7,53,54].

**Figure 7:**
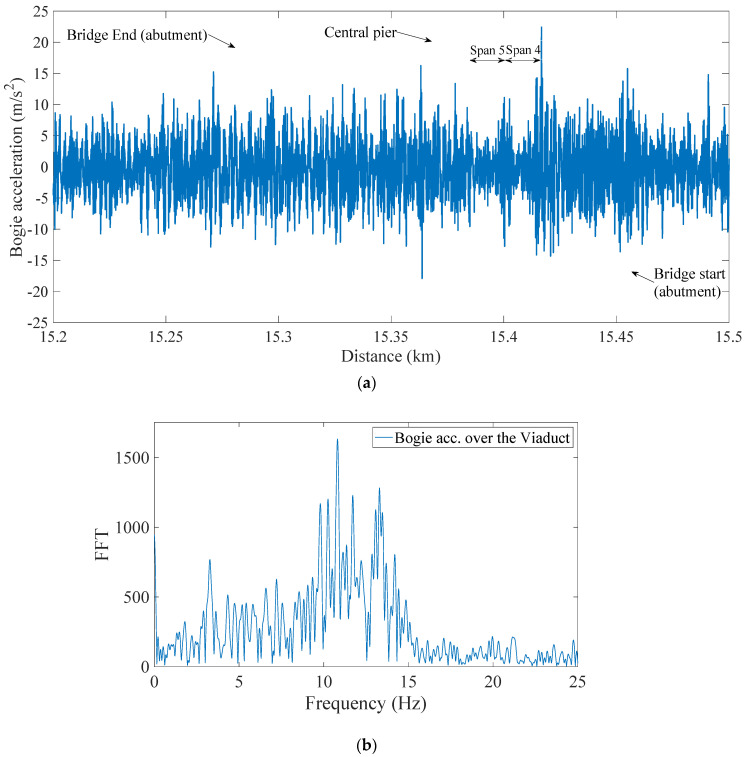
Bogie vertical acceleration in: (**a**) time domain, and (**b**) frequency domain (on−bridge data).

The train speed varied significantly between runs in the range, 85–120 km/h [7]. A sample of bogie vertical accelerations is presented in Figure 7. It can be seen in Figure 7b, which is the FFT of the on-bridge acceleration data, that the signal contains many frequencies, which may be associated with the train properties, the bridge/track properties or both. The train frequencies tend to have higher amplitude in the signal, which can make it difficult to estimate bridge frequencies. In this paper, a time-frequency domain analysis using EEMD based HHT is applied to estimate the natural frequencies of each span of the viaduct.

## 4. Analysis of Indirect Measurements

### 4.1. EEMD-Based HHT Analysis

**Figure 8:**
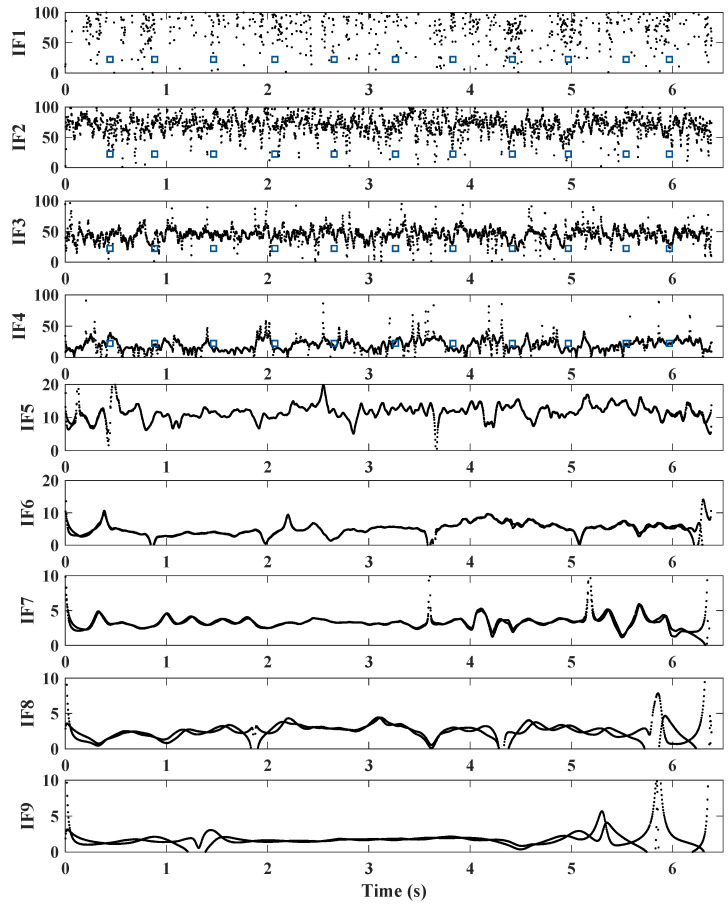
IFs (in time-frequency domain) of an acceleration signal from the instrumented train (dashed lines show the pier locations).

An EEMD-based HHT is applied to the drive-by acceleration signals (recorded from the instrumented train). To test the approach, a raw acceleration signal is decomposed into IMFs using the EEMD technique. The amplitude of the added noise and the number of iterations are determined using recommendations from the literature [49,50,52], and ten IMFs are calculated. In total, 25 iterations with an amplitude of 30% added noise are used in this analysis. These IMFs are converted into the time-frequency domain using the HT, resulting in the plots of IF. These IFs represent the changes in each IMF frequency as the train traverses the bridge. Figure 8 illustrates a result from the EEMD-based HHT for a single run of the train. It can be seen that IFs 1–4 contain higher frequencies (more than 25 Hz) as compared to the first natural frequencies. IFs 5–6 contain frequencies in the range between 5 and 15 Hz, which is closer to the natural frequencies of all the spans. IF 6, in particular, has frequencies in the range, 5 to 10Hz, which includes all bridge first natural frequencies (which lie between 6 Hz and 9 Hz). IFs 7–10 represent lower frequencies in the signal, that are not related to the bridge frequencies. 

**Figure 9:**
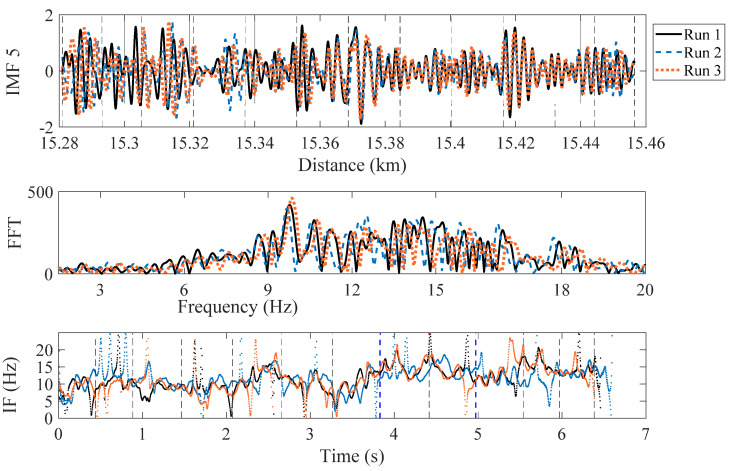
IF-5 and FFT calculated from IMF 5 (showing a range of frequencies closer to the spans natural frequencies) (red dashed line = replaced pier; blue dashed lines = boundary between the new and the other spans).

**Figure 10:**
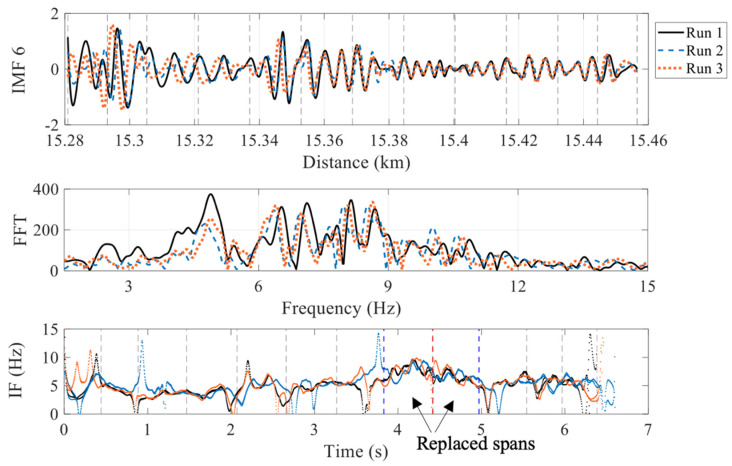
IF-6 and FFT calculated from IMF 6 (showing a range of frequencies closer to the spans natural frequencies) (red dashed line = replaced pier; blue dashed lines = boundary between the new and the other spans).

**Figure 11:**
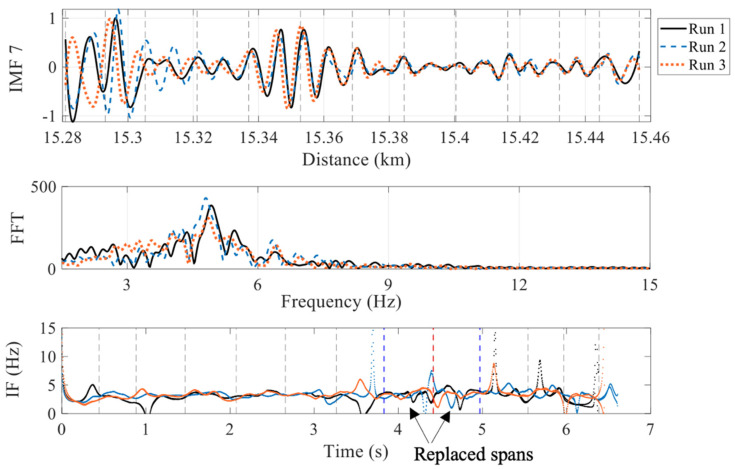
IF-7 and FFT calculated from IMF 7 (showing a range of frequencies closer to the spans natural frequencies) (red dashed line = replaced pier; blue dashed lines = boundary between the new and the other spans).

IMFs 5, 6 and 7 along with their FFTs and IFs are illustrated in Figure 9, Figure 10 and Figure 11, respectively. For checking the repeatability of the analysis, results from three runs are plotted in these figures. The FFTs and IFs of IMF 5 in Figure 9 show frequency components in this signal that lies in a range of 10–17 Hz, which are higher than the range predicted for the first natural frequencies of the spans. The FFTs of IMFs 6 (Figure 10) show that there are multiple frequencies in the range of 5–9 Hz, which are mixed together because of their proximity. The IF 6, on the other hand, shows that the frequencies change with time, especially in between 3.8 s and 5 s, where a modest increase in frequency is evident. This is the region where two spans and a pier have been replaced with stiffer components, which may explain the increase in the IF frequencies. This result may be used to estimate the bridge natural frequencies, or at least a pattern of changing bridge frequencies between spans. Although there may be a contribution from different train masses and velocities [56], the overall pattern of the frequencies may indicate changes in the span frequencies. The FFTs of the IMFs 7 (Figure 11) illustrate lower frequencies (lower than 5 Hz), that are not close to the bridge natural frequencies and therefore are not considered in this study.

### 4.2. Results and Discussion

**Figure 12:**
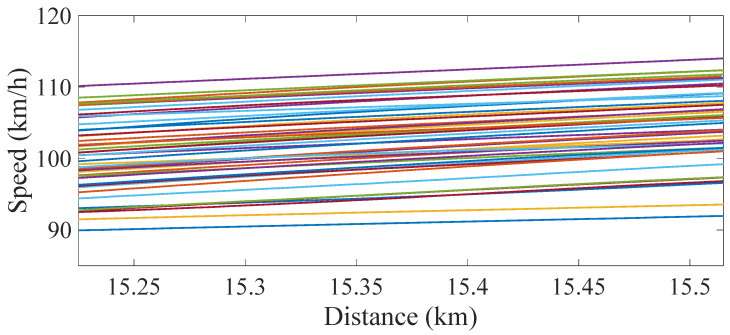
Speed of the train runs on the bridge-the distance on the X axis is from a reference point about 15 km before the bridge.

In this paper, EEMD-based HHT is used to identify the IFs from indirect acceleration measurements. As seen in Section 3, the first natural frequencies of the replaced bridge spans are higher than the frequencies of the original longer spans. In this section, IMF 6 is chosen to be used for estimating the pattern of changing frequencies in a multiple span bridge. Since the frequencies must be consistent for each span, an average of IFs for each individual span is considered. The train bogie accelerations from 41 train runs are used in this study to verify the efficacy of the proposed approach. The speed of each train changes as it traverses the bridge (mostly seen to increase in magnitude; see Figure 12) [7]. The details of the velocity effect on the train bogie accelerations are presented in [7].

**Figure 13:**
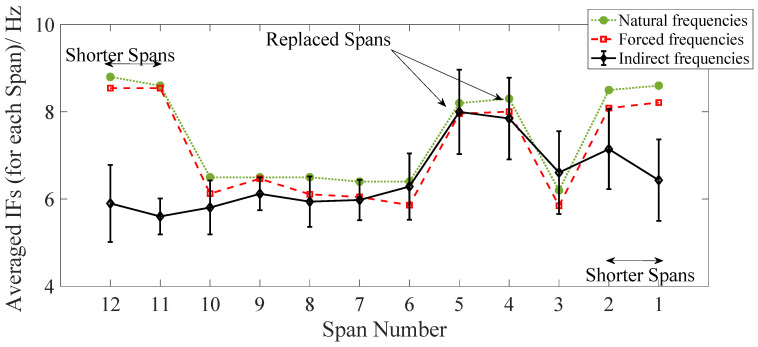
Averaged IFs of each Span (from IMF 6) from the indirect measurements (compared to the direct measurements).

**Figure 14:**
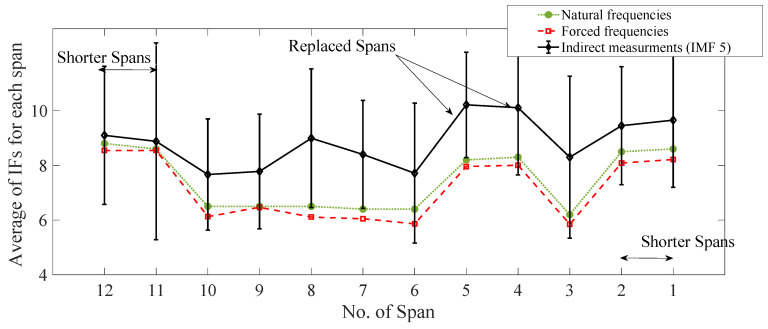
Averaged IFs of each Span (from IMF 5) from the indirect measurements (compared to the direct measurements).

Figure 13 shows the average of each span’s IFs (calculated using the IMF 6 measurement) estimated from the indirect measurements. The first natural frequencies measured directly from the free and the forced vibration measurements (Section 3) are also shown (dotted and dashed lines, respectively) for comparison. For the indirect measurements, as the results are collected from multiple runs, error bars are plotted using a mean and (+/−) two standard deviations (representing more than 95% of the total results). It can be seen in Figure 13 that the average of the IFs for each span approximately follows the pattern from the direct measurements of the first natural frequencies, especially where the replaced spans are located (Spans 4 and 5). In addition, the natural frequencies estimated from the indirect measurements are closer to the frequencies estimated from the forced vibrations. This is an important finding showing the shift in the estimated frequencies due to the presence of the train. This change in frequencies is due to the fact that the added mass effect of the train is present in both cases of drive-by and forced vibration, which resulted in a frequency shift compared to the free vibration scenario. In addition, for the spans which are equal in length, the consistency in frequencies around the unreplaced spans is well detected by the drive-by approach and follows the same pattern as the direct measurements. However, the results for the shorter spans are not accurate which may be caused by several reasons, e.g., mode mixing or the edge effects that exist in the HHT process. In order to evaluate the impact of mode mixing, the averaged IFs are re-calculated using IMF5 and are compared to the direct frequencies in Figure 14. This figure shows that the frequencies estimated using IMF5 are highly spread around the mean value for all the spans (compared to Figure 13), which means the results are less reliable compared to IMF6. However, the IFs from IMF5 estimate the fundamental frequencies of spans 11 and 12 with reasonable accuracy, while the results for spans 1 and 2 are still not acceptable. It can be concluded that a single IMF calculated from the proposed approach in this study can be used for estimating the fundamental frequencies of the spans with similar lengths when the spans with different stiffness values can be clearly detected. However, when the span lengths are different, the problem becomes more challenging and in some cases, a different IMF can reveal the fundamental frequencies of those spans. In addition, it can be concluded from Figure 6 that some of the IFs estimated using HHT includes edge effects at the beginning and end of the signals which could result in inaccuracy in the process of frequency estimation as observed in Figure 13 and Figure 14. 

### 4.3. Energy Amplitude of the Signal

**Figure 15:**
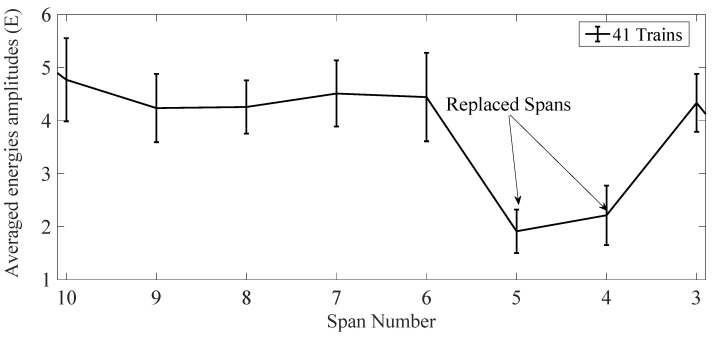
Sum of averaged energies per span using a mean and standard deviations from 41 train runs.

As reported in [7], the amplitude of the train acceleration signals for the replaced spans changes for the stiffer spans and the natural frequency, suggesting that the energy of frequencies for these responses will be lower for the replaced spans relative to the energies for the original spans. In this section, the amplitudes of the energy are assessed for each span. The purpose of this section is to determine how the energies of all the frequencies are affected and can be used for damage detection without having any prior knowledge of the spans’ natural frequencies. For that purpose, a sum of all the energies is considered. Each HT spectrum contains IF, energy amplitudes and phase angles for each IMF. An average of all the energies from each span is calculated for each IMF, which are then added together to determine the change of energies (in all the IMFs combined). Figure 15 shows the sum of averaged energies from each IMF for each span using the 41 trains runs with error-bars (mean +/− two standard deviations). As concluded in Section 4.3, for a multiple span bridge, it is better to employ the EEMD only for the spans of equal length. Therefore, only Spans 3–10 are used in this section. It can be seen in the figure that the averaged energy of the frequencies, combined from all the IMFs for each span, is less for the replaced spans. This indicates that the change in the span stiffness, not only changes the fundamental frequency of those spans, but also reduces the energy of the frequencies extracted from drive-by measurements using the signal processing tools (EEMD-based HHT). In this particular example, the higher stiffness to mass ratio of the replaced spans (negative damage) has resulted in a significant decrease in the energy amplitude of the IMFs. This is an important finding which can be used for developing novel damage indicators using drive-by approaches which can define global stiffness changes in multi span bridges. 

## 5. Conclusions

This paper presents a field investigation of direct and indirect/drive-by bridge health monitoring approaches for a multiple span viaduct. Two spans of the viaduct were replaced by stiffer beams in 2009 after they collapsed due to foundation scour. Acceleration responses, extracted from an instrumented train are used in this paper to (i) estimate the first natural frequencies of each span of the viaduct bridge, and (ii) detect the stiffness changes present in 2 out of 12 spans of the viaduct. Direct measurements with sensors installed on the bridge, are used to validate the results. The frequencies were estimated from the indirect measurements using the EEMD-based HHT technique. The HHT is a recent technique for indirect health monitoring. The EEMD instead of EMD is used to overcome the issue of mode mixing. This technique is used to extract natural frequencies of the spans with the help of IFs from the calculated IMFs. The HT energies are seen to be strongly affected by replacing (negatively damaging) the two spans. 

A summary of the results is as follows:

1. The EEMD approach can be employed with drive-by measurements to detect the fundamental frequency of each span of a multiple span bridge. However, some preliminary information about the bridge frequencies or their ranges is required for effective application of the proposed approach.
2. The natural frequencies estimated from indirect measurements are reasonably close to the direct measurements and both measurements follow the same pattern of frequency changes in the internal spans of equal length.
3. The results from forced vibrations (compared to free vibrations) are closer to the ones obtained from drive-by approach which is due to the added-mass effect of the crossing train.
4. The proposed approach shows reasonable results when is used for comparing the frequencies of the spans with same length, while it becomes more challenging for shorter spans.
5. The energy of the frequencies are seen to decrease in the two replaced spans. Therefore, it can be used as a damage indicator for loss of global stiffness in future studies.

## Figures and Tables

**Table 1:** The 1st Natural Frequencies of each span.

	The Fundamental Frequency (Hz)											
Span No.	1	2	3	4	5	6	7	8	9	10	11	12
Span length (m)	12.2	12.2	15.85	15.85	15.85	15.85	15.85	15.85	15.85	15.85	12.2	12.2
Free vibration	8.6	8.5	6.2	8.3	8.2	6.4	6.4	6.5	6.5	6.5	8.6	8.8
Forced vibration	8.2	8.1	5.8	8.0	8.0	5.9	6.0	6.1	6.5	6.1	8.5	8.5
